# Impact of surgical care pathways on efficiency and outcomes in orthopedic operating rooms: a historical control study

**DOI:** 10.1186/s12893-025-03135-w

**Published:** 2025-08-25

**Authors:** Tao He, Xiaojun Zhu, Guanghao Chi, Huan Chen, Min Hao, Xin Huang, Guanrong Wang

**Affiliations:** 1 College of Health Management, Shanghai Jian Qiao University, Shanghai, 201306 People’s Republic of China; 2 Operating Room, The Affiliated Hospital of Qingdao University, Qingdao, Shandong Province 266000 People’s Republic of China; 3 Department of Orthopedics, Hanzhong Central Hospital, Hanzhong, Shaanxi Province 723000 People’s Republic of China

**Keywords:** Care pathway, Orthopedics, Operating room, Surgical site infection, Patient satisfaction

## Abstract

**Objective:**

The impact of surgical care pathways (CP) on efficiency and patient outcomes in orthopedic operating rooms (OR) is unclear, and we aim to optimize nursing strategies and enhance service quality.

**Methods:**

From July 2019 to June 2024, 3,836 patients undergoing orthopedic surgery at a single Grade 3 Chinese hospital were retrospectively analyzed and divided into two equal groups, with or without CP (1,918 patients in each group). The effectiveness of CP was assessed by comparing surgical site infection (SSI) rates, pathogens, OR turnover times, and patient satisfaction. Statistical analyses included between-group comparisons and multivariate logistic regressions.

**Results:**

Baseline characteristics were balanced across groups (SMD < 0.1). Overall SSI rates were 2.1%, significantly lower in the CP group than in the Non-CP group (1.6% vs. 2.6%, *P* < 0.05), and translating to a 43% risk reduction (OR 0.57, 95% CI: 0.36–0.88, *P* < 0.05). Gram-positive bacterial infections notably decreased (OR 0.331, 95% CI: 0.093–0.959, *P* < 0.05). CP implementation also improved OR turnover times (95% CI: 0.504–0.839, *P* < 0.001) and patient satisfaction (95% CI: 1.038–2.301, *P* < 0.05). No significant differences were observed in other nursing quality indicators.

**Conclusion:**

Standardized CPs are effective in significantly reducing the incidence of postoperative SSIs, particularly those caused by Gram-positive bacteria, thereby enhancing infection control. Furthermore, these pathways improve OR operational efficiency and patient satisfaction, supporting reforms in OR management practices.

## Background

Surgical Care Pathways (SCPs), as a structured, multidisciplinary, collaborative care model, have demonstrated significant advantages in optimizing the perioperative management of patients [1]. With the rapid development of orthopedic surgical technology, it has put forward higher requirements for the quality of care in the operating room (OR). OR efficiency and quality of care of orthopedic surgery, as a key treatment for restoring patients’ motor function, directly affect patients’ prognosis and quality of life [2].

Existing studies have shown that there are obvious limitations in traditional care management models. The quality management system of OR care generally suffers from strong subjectivity and insufficient quantitative indicators. It is often difficult to comprehensively assess patients’ outcomes with a conventional quality control method [3]. This situation has prompted researchers to explore a more scientific system for assessing nursing care quality. The emergence of CPs has brought a new solution to this field, which has significantly improved the efficiency of care through standardized processes and interdisciplinary collaboration [4].

Several studies have confirmed the positive impact of CPs in orthopedic surgery. For example, the implementation of CP in hip replacement surgery reduced the average length of hospital stay from 13.3 days to 9 days and significantly reduced the complication rate [5]. A study by Lim et al. [6] further demonstrated that CP effectively improves surgical safety by optimizing teamwork and standardizing operating procedures. However, most of the existing studies focused on perioperative management, with relatively insufficient attention paid to intraoperative care aspects [5, 7].

It is worth noting that OR nursing staff face pressure from various sources. Eriksson et al. pointed out that factors such as complex surgical environments and tense team relationships may affect the effectiveness of CP implementation. A study by Bu et al. also found that these factors may indirectly affect patient outcomes [8, 9]. Therefore, it is clinically important to systematically assess the effectiveness of CP in the orthopedic OR.

This study used a historical-controlled design to comprehensively assess the impact of standardized CP on operational efficiency and patient outcomes in orthopedic ORs. The study focused on the following aspects: (1) primary outcome indicator: rate of surgical site infection (SSI); (2) subgroup analysis: characteristics of SSI. (3) secondary outcome indicators: surgical turnover time and patient satisfaction; confounders were controlled by methods such as propensity score matching (PSM) to ensure the reliability of the study results.

The innovations of this study are (1) the first systematic assessment of the comprehensive effect of surgical CP in orthopedic ORs; (2) the establishment of a scientific quantitative assessment system for nursing quality; and (3) the provision of an evidence-based basis for optimizing OR management. The results of the study will help to promote the standardization process of orthopedic surgical care and provide new ideas for improving the quality of medical services.

## Methods

### Study design and participants

This study used a matched historical controlled study design, aiming to compare the changes in orthopedic surgical care quality indicators before and after the implementation of the standardized surgical CP. The study population consisted of patients undergoing orthopedic surgery during the study period and their registered nurses responsible for perioperative care. Inclusion criteria: all patients undergoing elective orthopedic surgery during the study period, and the surgical nurses involved in their perioperative care were included in the study. Exclusion criteria: patients who underwent emergency surgery, had a history of repeat surgery, or had serious comorbidities that could interfere with postoperative outcomes were excluded. Specifically, patients with a preoperative diagnosis of infection or severe sepsis, as well as patients with grade IV surgical incisions, were excluded from the analysis, based on the National Academy of Sciences’ stratified wound classification system [10, 11].

### Study setting

The study was conducted in a large general hospital in China with more than 1,900 beds covering more than 52 medical specialties and care provided by more than 2,500 professionals. The department of surgery is equipped with 20 ORs and a team of more than 70 specialist nurses supporting a wide range of surgical procedures, including orthopedics, urology, general surgery, and cardiac surgery. The department provides surgical services to over 30,000 patients annually. The data collection period was from July 1, 2019, to June 30, 2024, a time span that allowed us to comprehensively assess the long-term impact of CPs on the quality and efficiency of orthopedic surgical care. The study was approved by the Institutional Ethics Review Board (IRB) under number 2024-22. All individuals who participated in the study and agreed to the release of their identifying information or images signed a written informed consent form, and the research process strictly adhered to the ethical principles of the Declaration of Helsinki.

### Intervention

The control group adhered to a traditional, non-standardized CP with personalized care activities based on the individual patient’s needs, while the intervention group implemented a standardized CP to improve the quality of care through a systematic approach and teamwork [12].

### Staffing, training, and management of CP

OR nurses assume a variety of roles, ranging from administrative oversight to professional team leadership and direct patient care. Nurses at all levels are assigned specific responsibilities guided by a patient-centered care philosophy. Intraoperative CPs were guided by experienced team leaders and supported by members with at least one year of experience. A “standardized training” model has been implemented, which focuses on progressive professional development with a curriculum designed by the OR education secretary and tailored to different levels of expertise. This training system consists of three years of standardized training and two years of specialized training in the OR, covering basic system protocols, standardized procedures, crisis management, and specialized surgical procedures [12].

### Implementation of Surgical CP

The surgical CP has been carefully designed to enhance the efficiency of orthopedic surgery and improve patient prognosis. The pathway is divided into preoperative, intraoperative, and postoperative phases, each with standardized processes and individualized interventions to ensure high-quality care and patient safety [13, 14] (see Table 1).

**Table 1:** Standardized procedures for surgical care pathways

**Preoperative care**			
**Steps**	**Responsible person**	**Main activities**	**Timetable**
Patient Assessment	Physician/Nurse	Performs a thorough physical examination, medical history review, and laboratory tests to determine if the patient is healthy enough for surgery.	1–2 weeks before surgery
Risk Assessment	Doctors/Nurses	Use a standardized risk assessment tool to predict the probability of complications such as SSI and develop individualized preventive measures accordingly.	
Patient education	Nurse/Health Education Specialist	Explain in detail to patients and their families the surgical procedure, expected outcomes, postoperative recovery plan, and how to self-manage.	
Nutritional support	Dietitian	Provide nutritional guidance or supplements according to the patient’s specific situation to ensure that the patient is in the best condition for the surgery.	
Counseling for smoking and alcohol cessation	Psychologist/Nurse	For patients who smoke or drink alcohol, provide professional advice and support services to quit smoking and drinking.	4–6 weeks before surgery
Medication adjustment	Doctor	Adjust the medications being used according to the doctor’s advice, especially anticoagulants or other medications that may affect the safety of the surgery.	1 week before surgery
Multidisciplinary team meeting	Medical team	A multidisciplinary team, including surgeons, anesthesiologists, and nurses, is organized to discuss each patient’s treatment plan.	
Family Involvement	Social worker/nurse	Invite the patient’s family to attend a meeting to learn about the surgical procedure and key points of post-operative care to enhance the family support system.	
**Intraoperative care**			
**Steps**	**Responsible person**	**Main activities**	**Timetable**
Aseptic technique	OR nurses	Clean and treat surgical areas using highly effective disinfectants to ensure a sterile surgical environment.	30 min before the start of surgery to the end of surgery
Equipment check	Technical support staff/OR nurse	Confirm that all instruments and equipment required for the surgery are in good working condition, and have an emergency plan in case of accidents.	1 h before the start of surgery
Staffing	OR Supervisor	Rationalize staffing in the OR to ensure that each step of the process is taken care of by a dedicated person.	
Clarify the division of roles	Surgical team members	Avoid confusion by determining the specific responsibilities of each team member beforehand. For example, who is responsible for monitoring vital signs, who is responsible for documenting the surgical procedure, and so on.	30 min before the start of surgery
Real-time feedback mechanism	Surgical team members	Establish effective communication channels so that team members can exchange information in a timely manner and solve problems quickly.	The entire surgical procedure
Standard Operating Procedures (SOP)	Surgical team members	Follow pre-determined standard operating procedures to ensure that every step is performed to the highest standards.	Surgical Procedures
Body temperature management	Anesthesiologists/nurses	Appropriate measures are taken to maintain the patient’s body temperature, e.g., use of heating blankets, etc.	Throughout Surgery
Pain control	Anesthesiologist/nurse	Adopt a multimodal analgesic strategy to minimize the impact of postoperative pain on the patient.	Whole Surgery
Fluid Management	Anesthesiologist/Nurse	Accurately control the volume and rate of fluid infusion to prevent complications caused by too much or too little fluid.	Whole Surgical Procedure
**Post-operative care**			
**Steps**	**Responsible person**	**Main activities**	**Timetable**
Vital signs monitoring	Nurse	Continuously monitor the patient’s blood pressure, heart rate, respiratory rate, and oxygen saturation, among other key indicators.	Immediately after surgery until discharge
Pain management	Physician/Nurse	Adjust medication dosage according to the pre-established multimodal analgesia program to ensure patient comfort.	Immediately after surgery, before pre-discharge
Wound Care	Nurse	Regularly inspect surgical incisions to prevent infection and change dressings when necessary.	Immediately after surgery until discharge
Complication prevention	Doctors/Nurses	Encourage early mobility, use compression stockings or anticoagulants; provide antiemetic medications; enforce strict hand hygiene practices, and limit the number of visitors.	Immediately after surgery until discharge
Patient education and support	Social Worker/Nurse	Explain to patients and their families what to expect after discharge, including guidance on medication, arrangements for follow-up appointments, and handling of emergencies; provide psychological counseling or support group services.	Immediately after surgery until discharge
**Post-operative rehabilitation care**			
**Steps**	**Responsible person**	**Main activities**	**Timetable**
Rehabilitation Program Development	Rehabilitation Specialist/Doctor	Formulate an individualized rehabilitation training plan according to the patient’s specific condition and recovery; set short-term and long-term rehabilitation goals.	From the 1 st week after discharge
Physical Therapy and Functional Exercise	Physical Therapist	Instruct patients in appropriate exercise training, gradually increasing the intensity; teach home exercise methods.	
Nutrition and Lifestyle Adjustment	Dietitian/Health Consultant	Provide personalized dietary advice according to the patient’s physical condition; continue to emphasize the importance of smoking cessation and alcohol restriction.	
Psychological support	Psychologist/Social Worker	Provide psychological counseling or support group services to help patients cope with anxiety and stress after surgery.	
Regular review and assessment	Doctors/Nurses	Arrange regular reviews for patients to assess the progress of recovery and adjust the treatment plan.	Every 2–4 weeks after discharge

*OR* Operating Room

**Fig. 1:**
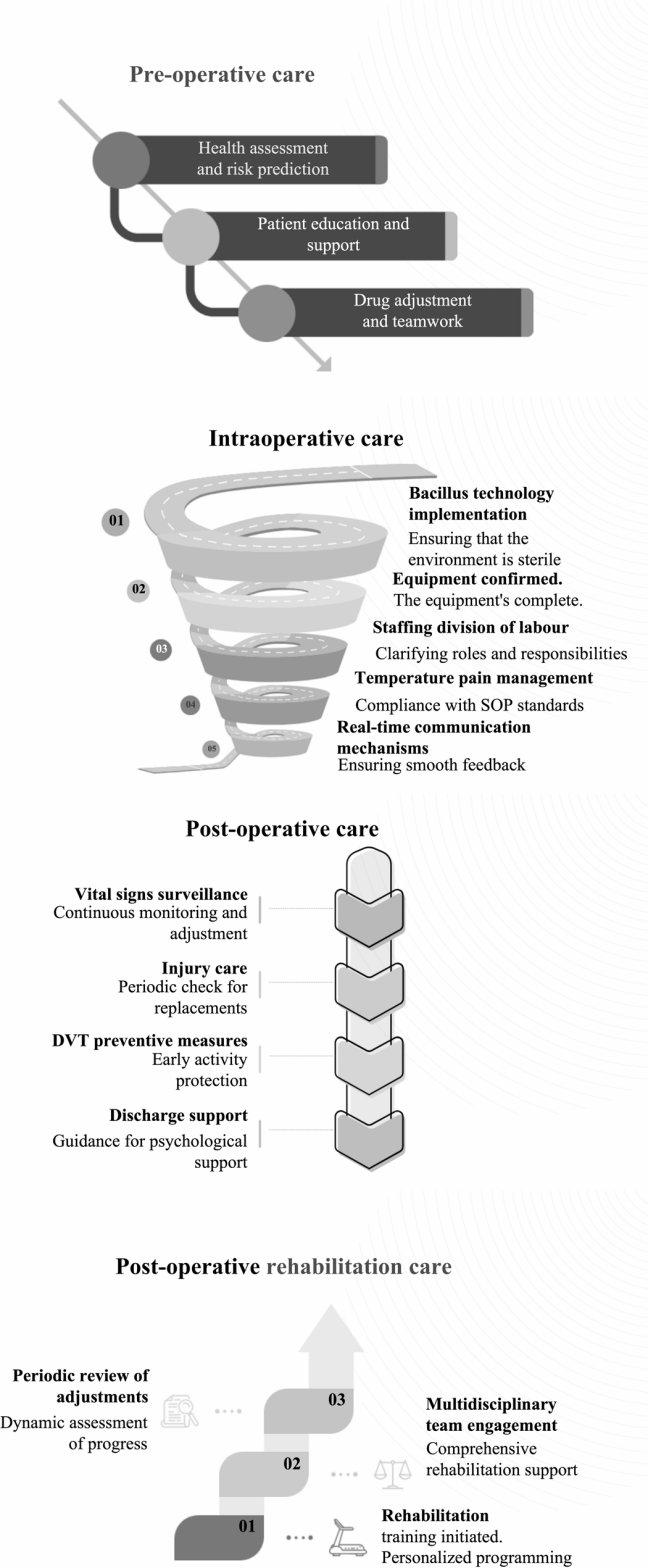
Flow chart of the surgical care pathway

### Preoperative care

#### Comprehensive assessment and individualized risk prediction

The preoperative phase of care begins with the implementation of a comprehensive patient assessment process. A healthcare team of physicians and nurses is responsible for performing a physical examination, medical history review, and laboratory tests to assess whether the patient has indications for surgery and to identify potential risk factors. This assessment is usually completed within 1–2 weeks prior to surgery. Subsequently, standardized risk assessment tools (e.g., ASA score, Charlson Comorbidity Index [15]) are used to predict the probability of SSIs and other complications, and individualized prevention strategies are developed accordingly.

#### Patient education and health behavior intervention

Next was the patient education session, in which nurses and health education specialists explained the surgical procedure in detail to patients and their families, including the purpose of the surgery, expected outcomes, postoperative recovery, and provided information on pain management, wound care, and rehabilitation training. The dietitian provides personalized dietary advice based on the patient’s nutritional status and prescribes nutritional supplements when necessary to ensure that the patient is in the best condition for the surgery. At the same time, patients are instructed in self-management, such as dietary adjustments and lifestyle changes. For smokers or alcohol drinkers, psychologists or nurses will provide support services for smoking and alcohol cessation 4–6 weeks before surgery.

#### Medication adjustment and preoperative preparation standards

Medication adjustments are an important part of preoperative care. The physician adjusts the medications being used, especially anticoagulants, one week prior to surgery based on the patient’s condition and surgical needs to minimize surgical risk. In addition, preoperative fasting and glycemic control criteria are confirmed: patients are allowed to drink water two hours before induction of anesthesia, but no solid food should be consumed for six hours prior to surgery to reduce insulin resistance and catabolic state. Routine sedation is not recommended for anxiety relief; acetaminophen, nonsteroidal anti-inflammatory drugs (NSAIDs), and gabapentin are recommended as part of multimodal analgesia. Anemia should also be assessed and corrected preoperatively, and iron or erythropoietin therapy should be given if necessary to reduce the need for allogeneic blood transfusions.

#### Multidisciplinary collaboration and family support

To ensure consistency and completeness of the treatment plan, a multidisciplinary team meeting is organized in which surgeons, anesthesiologists, nurses, and other relevant personnel discuss each patient’s treatment plan and clarify the preparations. Social workers or nurses invited patients’ families to participate in the meeting to help them understand the surgical process and key points of post-operative care, and to enhance the role of the family support system. Through these meticulous pre-operative preparation measures, not only is the safety and success rate of the surgery improved, but also a better perioperative experience is provided to the patients.

### Intraoperative care

#### Aseptic operation and equipment preparation

During the surgical execution phase, OR nurses strictly enforce aseptic techniques and thoroughly clean the surgical area using highly effective antiseptics, a process that lasts from 30 min before the start of the surgery until the end of the procedure. The technical support staff, in collaboration with the OR nurses, conducts a thorough inspection of all required instruments and equipment to ensure that they are in good working order and develops a contingency plan to deal with any unforeseen circumstances that may occur. The OR supervisor is responsible for rationalizing the division of labor among the staff to ensure that each step of the process is handled by a dedicated person, usually one hour before the start of the procedure.

#### Clarification of team roles and standardized procedures

Thirty minutes before the start of surgery, the surgical team members clarify their roles and responsibilities to ensure that each member is clear about his or her own tasks, such as monitoring vital signs and recording the procedure. Surgical procedures are customized based on the individual case, the surgeon’s experience, and the hospital’s facilities. During surgery, an effective communication mechanism is established to facilitate information sharing and rapid problem-solving among team members, and this real-time feedback mechanism is important for improving the safety and efficiency of the surgery. All team members strictly adhere to pre-established standard operating procedures (SOPs) to ensure that every step is of the highest standard.

#### Temperature management and analgesic fluid control

Anesthesiologists work closely with nurses to maintain normal levels of patient body temperature and take measures to prevent hypothermia, such as intravenous fluid warming and preheating equipment. At the same time, multimodal analgesic strategies, including epidural morphine, local block, or long-acting infiltrating anesthetics, were implemented to optimize postoperative pain management. Strict fluid management is performed during surgery, with precise control of the volume and rate of infusion and the use of balanced intravenous fluids to maintain isotonicity and prevent complications due to fluid imbalance. Nursing care at this stage is a key component in ensuring smooth surgery and patient safety.

### Postoperative medical care

#### Vital signs monitoring and analgesia management

During the postoperative care phase, the nurse is responsible for continuously monitoring the patient’s key vital signs, such as blood pressure, heart rate, respiratory rate, and oxygen saturation, to ensure that the patient remains stable during the postoperative recovery period. This process begins immediately after the patient’s surgery and continues until discharge. Doctors and nurses adjust medication dosages according to a pre-established multimodal analgesia program to ensure patient comfort and reduce pain and discomfort after surgery.

#### Complication prevention and incision care

Nurses regularly inspected surgical incisions to prevent infection and changed dressings in a timely manner. A series of measures were taken to prevent common complications: routine use of urinary catheters was avoided, and if needed, they should be removed within a few hours after surgery; routine use of wound drainage was not recommended, especially after short-segment lumbar fusion, to promote early recovery. Encourage patients to be active early and use compression stockings or anticoagulants to reduce the risk of deep vein thrombosis (DVT). Assess the risk of postoperative nausea and vomiting (PONV), use a variety of medications for prophylaxis, and instruct patients on dietary modifications to reduce symptoms. Strictly enforce hand hygiene practices and limit the number of visitors to minimize the chance of cross-infection.

#### Discharge guidance and psychological support

In addition, social workers and nurses explain to patients and their families what to expect after discharge, including guidance on medication, arrangements for follow-up consultation, and handling of emergencies. Psychological counseling or support group services are also provided to help patients cope with anxiety and stress after surgery. The goals of nursing care at this stage are to promote patients’ recovery, prevent complications, and lay the foundation for subsequent rehabilitative care.

### Post-operative rehabilitation care

#### Early feeding and individualized recovery plan

Post-operative rehabilitation care emphasizes early feeding to speed up the recovery process. Rehabilitation specialists and doctors develop individualized rehabilitation training plans based on the patient’s specific condition and recovery and set short- and long-term rehabilitation goals, such as regaining normal mobility and returning to work. These programs are usually initiated in the 1 st week after the patient is discharged from the hospital.

#### Exercise training and nutritional support

Physical therapists instruct patients in appropriate exercise training, gradually increasing the intensity, and teach home exercise methods to promote the recovery of daily mobility and physical function. To address the risk of thromboembolism, early mobility out of bed and the use of mechanical prophylaxis were encouraged, and the use of anticoagulants was considered on an individualized basis. Dietitians and health advisors provide individualized dietary advice based on the patient’s physical condition to promote wound healing and overall health.

#### Mental health and regular follow-up

Continue to emphasize the importance of smoking cessation and alcohol restriction to avoid compromising the recovery process. Psychologists and social workers provide psychological counseling or support group services to help patients cope with post-surgery anxiety and stress, and arrange counseling sessions to help patients develop a positive mindset. Doctors and nurses arrange regular reviews for patients, usually every 2–4 weeks, to assess the progress of recovery and adjust the treatment plan to prevent other complications. Rehabilitation outcomes are assessed using standardized tools and documented. Postoperative rehabilitation care is a critical phase in achieving functional recovery and improving quality of life.

The standardized nursing process is based on the standardized nursing care path formulated by the department. The responsible nurse cares for patients according to the care path table, which consists of a reference time for CP, an actual execution time for CP, the content of CP, an evaluation of effect, and the signature of the person who executed CP. Nurses provide continuous, dynamic, targeted, and standardized health education to patients from preoperative to postoperative stages based on the CP path. The implemented educational content is marked with a blue pen, while unimplemented content is marked with a red pen. The reason why the unimplemented content was not carried out must be recorded in the nursing record by the duty nurse [12].

### Outcome

The primary outcome metrics focused on the incidence of SSIs during hospitalization, the type of pathogen, and its incidence. Postoperative SSIs, as defined by the Centers for Disease Control and Prevention, involve infections of the skin, subcutaneous tissues, deep soft tissues, or any anatomical site within 30 days after surgery that are diagnosed as SSIs by the surgeon or attending physician and are included in the study period to ensure that the surveillance methodology remains consistent across the two study periods. Secondary outcome indicators covered surgical turnover time, patient satisfaction scores with nurses and physicians, and other indicators sensitive to quality of care. Surgical turnover was defined as the time interval between the end of one surgery and the start of the next, usually including the time required to clean the OR, prepare equipment, and rearrange. A positive event was defined as a turnover time of more than 30 min between surgical procedures [16]. Patient satisfaction scores were collected by means of questionnaires, and patient satisfaction ratings of surgical and related services were usually quantified using a Likert scale (e.g., 1–5), which included, but were not limited to, the attitude of healthcare professionals, surgical outcomes, hospitalization environment, pain management, and transparency of information [17].

### Data collection

Patient information and care data, including the type of procedure, duration, SSI events, patient outcomes, and caregiver feedback, are carefully collected through electronic medical record systems and OR records.

### Validity and reliability

In this study, we used validation tools with good reliability to improve the validity and reliability of the data [16]. We have extensive experience in care management, and quality indicators were available throughout the data collection period. Consistency was maintained using quality indicators and procedures prior to the study design plan, including procedure-related complications, procedure-related quality indicators, and patient satisfaction. The validity and reliability of the data collection instruments were ensured by validating the accuracy of data entry and using standardized assessment tools and scales. All data were scrutinized, and data entry was performed twice to eliminate input errors. Confounders were controlled for using PSM methods to ensure baseline balance between CP and non-CP periods.

### Sample size calculation

The sample size was calculated to be 3,836 patients (1,918 in each group) using G*Power ver. 3.1.9.4 Software. Assuming a baseline SSI rate of 2.6%, with an expected reduction to 1.6% post-CP implementation, a two-sided test was used with an alpha level of 0.05 and a desired power (1 - β) of at least 80% [18].

### Statistical analysis

Patients were categorized according to the care management they received during the non-CP and CP periods. Categorical variables were presented as numbers and percentages, whereas continuous variables were described in detail by mean (standard deviation) or median (interquartile range). To compare SSI rates and baseline characteristics between the CP and non-CP groups, standardized mean differences (SMDs) were used. In this study, we used a logistic regression model to estimate propensity scores and a nearest-neighbor matching strategy (minimum caliper value of 0.1) for matching. The multivariate model included baseline characteristics such as age, gender, comorbidities, and type of surgery. Initial screening identified significantly relevant variables by univariate analysis (*P* < 0.2), followed by stepwise regression to identify key predictor variables and to consider significant interaction effects. Hypothesis testing included checking for linear relationships between continuous variables and logit-transformed dependent variables, independence of observations, multicollinearity (Variance inflation factor (VIF) > 10 indicates a serious problem), and outliers or high-leverage points in the residual distribution. The overall model fit was verified using the Hosmer-Lemeshow goodness-of-fit test, and VIF values were calculated for each variable to remove excessive values or transformed variables. For missing data, a full case analysis was first performed, and then the Multivariate Imputation by Chained Equations (MICE) algorithm was used to generate multiple filler datasets and merge the results through the multiple filler method [19, 20]. To further validate the effect of CP, we calculated the ratio of patients’ ratios and their 95% confidence intervals (CIs) before and after pairing by multivariate logistic regression analysis. The comparisons of secondary outcome indicators were analyzed using the Fisher exact test or chi-square test for count data, and the Mann-Whitney U test or t-test for numerical variables, as appropriate. All statistical analyses were done through SPSS 26 software to ensure robust and reliable results.

## Results

By making a detailed comparison of the baseline characteristics of the two groups of patients, including variables such as age, gender, comorbidities, and type of surgery, the results of this study showed that SMD of all covariates included in the analysis was less than 0.1, which indicated that the two groups were highly comparable with each other in terms of their demographics and clinical characteristics, and provided a good basis for subsequent comparative analyses (see Table 2).

**Table 2:** Baseline characteristics of the patients in the surgical group, pre- and post-propensity score matching between the CP group and non-CP group

Variable	CP Group (*N* = 1918)	Non-CP Group (*N* = 1918)	SMD
Female, n (%)	895 (46.66%)	883 (46.04%)	0.014
Age, mean (SD)	45.22 (13.36)	44.64 (12.33)	0.042
Diabetes, n (%)	179 (9.33%)	157 (8.19%)	0.040
Hypertension, n (%)	316 (16.48%)	306 (15.95%)	0.015
Surgical wound classification, n (%)^*^			0.017
Class I (clean)	1770 (92.28%)	1776 (92.60%)	
Class II (clean-contaminated)	54 (2.82%)	55 (2.87%)	
Class III (contaminated)	94 (4.90%)	87 (4.54%)	
Surgery type, n (%)			0.016
Trauma	472 (24.61%)	480 (25.03%)	
Joints	529 (27.58%)	526 (27.42%)	
Spine	690 (35.97%)	692 (36.08%)	
Others	227 (11.84%)	220 (11.47%)	

*CP* Care Pathway, *SMD* Standardized Mean Difference

^*^The current surgical wound classification system of the National Academy of Sciences, which stratifies wounds into 4 categories (clean, clean-contaminated, contaminated, and dirty), one case of Class IV (dirty) was excluded according to the exclusion criteria

### Primary outcome

Overall, the SSI rate during the study period was 2.1%. Further analysis showed that the SSI rate in the non-CP group was 2.6%, while it was significantly reduced to 1.6% in the CP group (*p* < 0.05). After adjusting for potential confounders through a multifactorial logistic regression model, the results showed that implementation of CP reduced the risk of SSI by 43% (OR = 0.57, 95% CI: 0.36–0.88, *P* < 0.05) (see Table 3). This result suggests that standardized CP has a significant effect in reducing postoperative infections and supports its value as a surgical quality improvement tool.

**Table 3:** Multifactorial logistic regression analysis of surgical site infections in CP and non-CP groups

Variables	Matched	
	OR Ratio (95% CI)	Significance of *P* value
CP Group (*N* = 1918)	0.57 (0.36 to 0.88)	< 0.05
Female	0.71 (0.43 to 1.14)	
Age	1.00 (0.99 to 1.01)	
Diabetes	1.07 (0.50 to 2.09)	
Hypertension	0.98 (0.49 to 1.82)	
SWC Class I*	1	1
SWC Class II	5.48 (2.80 to 10.42)	
SWC Class III	7.03 (4.00 to 12.39)	
Bones and Joints	1	1
Joints	0.04 (0.00 to 0.19)	
Spine	0.24 (0.10 to 0.49)	
Others	1.93 (1.12 to 3.36)	

*CP* Care Pathway, *SWC* Surgical Wound Classification, *CI* Confidence Interval

*The current wound classification system of the National Academy of Sciences in the United States, which classifies wounds into four categories (clean, clean-contaminated, contaminated, and dirty), excludes one case of category IV (dirty) based on exclusion criteria. Surgical Wound Classification Class I (Clean) is used as a reference category, and Bones and Joints is used as a reference category for surgery types

In order to gain a deeper understanding of the mechanisms by which CPs affect SSIs, we further analyzed the pathogen types of patients with SSIs. The results showed that 12 (0.63%) patients in the non-CP group had infections caused by Gram-positive bacteria, whereas only 4 (0.21%) were found in the CP group, a statistically significant difference (OR = 0.331, 95% CI: 0.093–0.959, *P* < 0.05) (see Table 4). This suggests that standardized CP may be effective in reducing the risk of specific types of infections, especially those caused by gram-positive bacteria, by enhancing perioperative asepsis, standardizing antibiotic use, or improving consistency of care. This subgroup analysis further reinforces the positive role of CP in infection control.

**Table 4:** The pathogen of surgical site infections during and after CP in orthopedic surgery

Pathogen	CP Group (*N* = 1918, %)		Non-CP Group (*N* = 1918, %)		Significance of *P* value
Gram-positive bacteria	4	0.21%	12	0.63%	< 0.05
Enterococcus faecium	0	0.00%	2	0.10%	
Enterococcus faecalis	0	0.00%	2	0.10%	
Staphylococcus aureus	3	0.16%	3	0.16%	
Staphylococcus haemolyticus	0	0.00%	2	0.10%	
Staphylococcus saprophyticus	0	0.00%	1	0.05%	
Streptococcus agalactiae	1	0.05%	0	0.00%	
Streptococcus pyogenes	0	0.00%	2	0.10%	
Gram-negative bacteria	17	0.89%	25	1.30%	
Acinetobacter baumannii	2	0.10%	4	0.21%	
Escherichia coli	2	0.10%	6	0.31%	
Klebsiella pneumoniae	3	0.16%	6	0.31%	
Moraxella catarrhalis	4	0.21%	4	0.21%	
Proteus mirabilis	1	0.05%	0	0.00%	
Pseudomonas aeruginosa	1	0.05%	0	0.00%	
Burkholderia cepacia	0	0.00%	1	0.05%	
Stenotrophomonas maltophilia	2	0.10%	4	0.21%	
Fusarium	1	0.05%	0	0.00%	
Yeast	1	0.05%	0	0.00%	
Fungi	9	0.47%	10	0.52%	
Aspergillus	0	0.00%	1	0.05%	
Candida	4	0.21%	5	0.26%	
Candida albicans	1	0.05%	2	0.10%	
Candida tropicalis	1	0.05%	0	0.00%	
Candida parapsilosis	1	0.05%	2	0.10%	
Trichosporon	2	0.10%	0	0.00%	

*CP* Care Pathway

### Secondary outcome

In addition to infection control, this study also assessed the impact of the CP on the operational efficiency of the orthopedic OR and patient experience. The results showed a significant decrease in OR turnover time from the baseline level in the non-CP group after the implementation of CP, with an OR of 0.653 (95% CI: 0.504–0.839, *p* < 0.001), which translates into a reduction in turnover time of more than 30 min by approximately 35%. This improvement suggests that the CP helped to optimize the surgical process, reduce preparation time, and enhance overall OR operational efficiency. In addition, the results showed that patient satisfaction was significantly higher in the CP group than in the non-CP group, with an OR of 1.543 (95% CI: 1.038–2.301, *p* < 0.05), implying a 54.3% improvement in patient satisfaction. This result not only reflects the effectiveness of the CP in improving the quality of service but also indicates a higher level of patient acceptance of the consistency and structure of the entire surgical procedure. In terms of other secondary quality of care indicators (e.g., mean length of stay, readmission rate, postoperative analgesia management, etc.), the difference between the two periods did not reach statistical significance (see Table 5).

**Table 5:** Secondary outcome comparisons of care-sensitive quality indicators between the CP and non-CP periods

Sensitive Quality Indicator	CP Group (*N* = 1918)	Non-CP Group (*N* = 1918)	χ²	Significance of *P* value
Patient Safety Indicator				
Confirmation of Surgical Procedure Name, n (%)	1918 (100)	1918 (100)		
Surgical Instrument Inventory Rate, n (%)	1918 (100)	1918 (100)		
Surgical Specimen Check Rate, n (%)	1918 (100)	1918 (100)		
Postoperative Surgical Equipment Check Rate, n (%)	1918 (100)	1918 (100)		
Acute Pressure Sore Rate During Surgery, n (%)	4.0 (0.21)	3.0 (0.17)	0.32	
Retained Surgical Foreign Object Rate, n (%)	0.0 (0.0)	0.0 (0.0)		
Perioperative Medication Usage Rate, n (%)	1918 (100)	1918 (100)		
Efficiency Indicator				
Surgical Start Time Delay Rate ^a^, n (%)	186.0 (9.72)	188.0 (9.8)	3.51	
Turnover Rate per Surgery ^b^, n (%)	78.0 (4.09)	103.0 (5.38)	11.29	< 0.001
Crisis Indicator				
Crisis Training Completion Rate ^c^, n (%)	6.0 (0.0033)	12.0 (0.0063)	0.38	
Patient Experience Indicator				
Patient Satisfaction, Median (Interquartile Range)	79 (75.8–79.9)	82.1 (79.7–87.1)	4.62	< 0.05

*CP* Care Pathway

^a^denominator of 614 first surgery days per year

^b^denominators of 946 and 1054 for non-CP and CP periods, respectively; positive events are defined as cases with an interval between two surgeries over 30 min

^c^data are the annual performance rates (≥ 80 points out of a total score of 100 points) of 13 nurses in the CP group and 22 nurses in the non-CP group

## Discussion

This study evaluated the implementation of a standardized surgical CP in an orthopedic OR and showed that this strategy significantly reduced the incidence of SSIs, increased OR turnover efficiency, and improved patient satisfaction. Specifically, after CP implementation, the overall SSI rate decreased from 2.6 to 1.6%, with a 1% point reduction in absolute risk and a 43% reduction in relative risk (*P* < 0.05), a result consistent with several studies. For example, Ana et al. found that structured perioperative management in hip arthroplasty reduced the SSI rate and operating time [21], and special orthopaedic geriatrics reported similar results in both hip and knee replacement [22]. In addition, subgroup analysis showed that the rate of Gram-positive bacterial infections was significantly lower in the CP group than in the non-CP group, suggesting that the CP effectively controlled the transmission of specific pathogens by standardizing aseptic practices and optimizing antibiotic use.

Analyzed clinically and economically, although the absolute reduction in SSIs was only 1%, in the context of thousands of orthopedic surgeries per year, this difference implies dozens of infections prevented, which is of great public health significance. Each case of SSI prevention saves approximately $10,000 to $20,000 in healthcare costs, including direct costs such as antibiotic treatment, extended hospital stays, and additional surgeries, as well as indirect costs such as loss of productivity and damage to the hospital’s reputation due to delays in patient recovery [23]. Thus, CP not only enhances patient safety but also demonstrates favorable cost-effectiveness at the economic level.

In further analysis, we found the implementation of CP significantly improved OR efficiency as evidenced by a 35% reduction in surgical turnover time of more than 30 min. This suggests that the CP helped to optimize process articulation, reduce waiting times, and improve resource utilization. Meanwhile, patient satisfaction improved by 54.3% (*P* < 0.05), reflecting patients’ recognition of the consistency of the entire surgical process, the quality of communication, and the service experience. These results were not only statistically significant but also had a substantial impact on hospital operational management and patient experience.

It is worth noting that although positive changes were observed in multiple dimensions in this study, some care-sensitive indicators did not show significant differences. For example, changes in dimensions such as crisis management training completion rates were not significant. This may be related to the limitations of the measurement methodology or may reflect the fact that some improvements in quality of care require longer or more intensive interventions to become apparent. In addition, as this study is a historical control design, there is a certain risk of selection bias, and although we used PSM and multiple infill methods to mitigate the effects of missing data and confounding factors, further validation of causality in future randomized controlled trials is needed.

Successful implementation of CP is highly dependent on the hospital-specific environment, including staffing, organizational culture, resource allocation, and quality management capabilities [24]. For example, hospitals with adequate resources are more likely to promote pathways on the ground, whereas organizations with strained human resources or inadequate information systems may face challenges. In addition, patient heterogeneity (e.g., age, underlying disease) can affect the effectiveness of pathways, with older patients or those with chronic conditions often requiring more individualized adjustments. Similarly, different types of surgeries and levels of team expertise determine the quality of pathway implementation. The policy support of the healthcare system, the healthcare payment mechanism, and the socio-economic context are also important factors influencing promotion [25].

Global studies have been conducted to show that prevention of SSIs is a multistage, systematic task that must incorporate preoperative preparation, intraoperative asepsis, and postoperative management [26]. Surgical CPs, as a structured management tool, can integrate the standard operating procedures recommended by the World Health Organization and ensure seamless integration and clarity of responsibilities in each step of the process, thus improving execution and consistency [27]. Jimenez-Martinez et al. noted that multimodal interventions are effective in reducing SSIs and that CP is an effective platform for achieving such an integrated strategy [28]. Recent studies also emphasized that the standardization of clean-aseptic practices is a key factor in the control of SSIs [5, 29] and was particularly prominent in reducing S. aureus infections [30], which further supports the findings of this study.

Additionally, a reduction in surgical procedure turnover time has also been identified as an important component in improving efficiency in the OR [31]. Sapon et al. noted that the involvement of surgical specialists can help to reduce turnover time [32], whereas a study by Shackelford et al. demonstrated that standardized care teams are effective in improving surgical transfer efficiency [33]. Our findings are consistent with this, suggesting that the CP significantly improved the overall operational efficiency of the OR through enhanced teamwork and process standardization.

In the past, orthopedic research has been more focused on basic and clinical techniques, but the fact is that advances in nursing techniques have been particularly important [34–36]. The strengths of this study are the long-term follow-up (July 2019 to June 2024), large sample size (*n* = 3,836), and systematic quality control measures, which resulted in high internal validity of the findings, especially for the control of Gram-positive bacterial infections. However, there are still some limitations. There are several limitations to this study. First, as a historical controlled study, although we used PSM and multiple fillers to reduce bias, confounding factors such as the influence of the COVID-19 pandemic in this period or the learning curve of CP could not be completely excluded, and causal inferences need to be further validated by prospective randomized controlled trials (RCTs). Second, in terms of surgical efficiency assessment, relying on a single turnover time index and lacking a more accurate surgical time recording system, there are some limitations to its sensitivity and representativeness. Third, this study was a single-center retrospective design with limited external generalizability, and some secondary quality indicators did not show significant improvement, suggesting that certain nursing interventions may require longer or stronger efforts to show effects. In addition, there were practical challenges in the implementation process, such as inconsistent implementation standards due to nurse rotation between different units, as well as hospital resource allocation, organizational culture, and other factors affecting the effectiveness of pathway implementation. Patient heterogeneity, differences in types of surgery, and healthcare policy context also add to the complexity of cross-institutional replication. Therefore, localization factors should be taken into account when interpreting the results, and future studies should be conducted in diverse settings, combining quantitative and qualitative methods to explore the optimal implementation strategies for CPs.

In summary, this study confirms the significant effectiveness of implementing standardized CPs in orthopedic ORs in reducing SSI rates, enhancing surgical efficiency, and improving patient experience. These results provide an empirical basis for surgical quality improvement and serve as a reference for replicating such interventions in other healthcare organizations. Future studies should further explore the applicability and sustainability of CP in different types of surgeries, hospital sizes, and national healthcare systems, and validate its long-term effects through RCTs. Meanwhile, it is recommended to strengthen the professional training and team building of nursing staff and to promote the development of advanced practice nursing models to better adapt to the advances in modern surgical techniques and the rising needs of patients.

## Data Availability

Data are available from the institutional medical chart database with relevant approval. However, restrictions apply to the availability of these data, which were used under license for this study and thus are not publicly available. The datasets used and/or analysed during the current study are available from the corresponding author on reasonable request.

## Abbreviations

CP: Care Pathway
OR: Operating Room
PSM: Propensity Score Matching
SSIs: Surgical Site Infections
SD: Standard Deviation
IQR: Interquartile Range
SMDs: Standardized Mean Differences
CI: Confidence Interval
